# Evolution of breast cancer management in Ireland: a decade of change

**DOI:** 10.1186/1471-2482-9-15

**Published:** 2009-09-18

**Authors:** Helen M Heneghan, Ruth S Prichard, Amanda Devaney, Karl J Sweeney, C Malone, Ray McLaughlin, Michael J Kerin

**Affiliations:** 1 Department of Surgery, National University of Ireland Galway, Ireland; 2 National Breast Cancer Screening Programme, Galway University Hospital, Galway, Ireland

## Abstract

**Background:**

Over the last decade there has been a paradigm shift in the management of breast cancer, subsequent to revised surgical oncology guidelines and consensus statements which were derived in light of landmark breast cancer clinical trials conducted throughout the latter part of the 20th century. However the sheer impact of this paradigm shift upon all modalities of treatment, and the current trends in management of the disease, are largely unknown. We aimed to assess the changing practices of breast cancer management over the last decade within a specialist tertiary referral Breast Cancer Centre.

**Methods:**

Comparative analysis of all aspects of the management of breast cancer patients, who presented to a tertiary referral Breast Cancer Centre in 1995/1996 and 2005/2006, was undertaken and measured against The European Society for Surgical Oncology guidelines for the surgical management of mammographically detected lesions [1998].

**Results:**

613 patients' case profiles were analysed. Over the last decade we observed a dramatic increase in incidence of breast cancer [>100%], a move to less invasive diagnostic and surgical therapeutic techniques, as well as increased use of adjuvant therapies. We also witnessed the introduction of immediate breast reconstruction as part of routine practice

**Conclusion:**

We demonstrate that radical changes have occurred in the management of breast cancer in the last decade, in keeping with international guidelines. It remains incumbent upon us to continue to adapt our practice patterns in light of emerging knowledge and best evidence.

## Background

Breast cancer is the commonest female malignancy in the developed world, and its incidence continues to rise. The Irish National Cancer Registry predicts that by 2020 there will be approximately 5000 new cases per annum in Ireland [1]. Over the past two decades awareness of the disease has increased dramatically, and in conjunction with increasing knowledge and understanding the management of breast cancer has evolved. Following several landmark clinical trials conducted throughout the mid to latter part of the 20th century [Veronesi, Sarrazin, Fisher, 2-7], the mid-1990s saw the publication of numerous sets of guidelines advocating a less radical surgical therapeutic approach, when combined with adjuvant radiotherapy, encouraging increased use of systemic therapies for estrogen responsive tumours, emphasising the importance of multidisciplinary team management, and highlighting a growing awareness of the psychosocial needs of breast cancer patients. The European Society for Surgical Oncology put forth recommendations which included specific targets and outcome measures, and a proposed framework for surgical quality assurance in breast cancer units, and recommended that these guidelines be reviewed and modified in 3 years in light of new knowledge [2]. Since the publication of these guidelines there has been a paradigm shift in the management of breast cancer, towards less invasive diagnostic modalities and surgical approach, which has ameliorated both the physical and psychological morbidity for women with this disease. The introduction of breast cancer screening has changed the type and stage of disease at presentation, and poses specific therapeutic challenges. Newer more specific adjuvant therapies and protocols have been trialled and are in widespread clinical use. The emphasis on women's psychosocial health following breast cancer treatment has drawn attention to the aesthetic outcome and the potential benefits of immediate breast reconstruction. However the sheer impact of this paradigm shift upon all modalities of treatment and the current trends in management of the disease are largely unknown. We aim to assess the changing practices of breast cancer management over the last decade within a specialist tertiary referral Breast Cancer Centre.

## Methods

A comprehensive comparative analysis was performed of the management of breast cancer patients who presented to a tertiary referral Breast Cancer Centre in 2005/2006, with those who presented in 1995/1996. All patients diagnosed with breast cancer in our unit during these years were identified from a prospectively collected database. The demographic characteristics, clinical, operative and histopathological records and follow-up details were reviewed. We compared all new breast cancer cases from these two 24 month periods with respect to the following parameters: incidence, age at diagnosis, diagnostic procedure, histologic type and grade, nodal status, stage [TNM] and prognostic index, primary surgical therapeutic procedure, adjuvant therapy, and subsequent surgical procedures. The staging method for all patients, which was applied retrospectively for the purpose of this review, was the 2003 Revised TNM Staging System for Breast Cancer [3].

All data were analysed using the software package SPSS 15.0 for Windows. Both the number of observations and percentages are presented to describe categorical variables. Differences in management between the groups of patients were calculated by means of two-sample t-test for all two sample comparisons, and the chi square test for binomial comparisons when appropriate. All tests were two tailed and a p-value of <0.05 was assumed to represent statistical significance.

## Results

A total of 613 patients were identified for inclusion in this study. Patients in both groups had similar demographic characteristics, including gender, and age at diagnosis [Table 1]. The overall incidence of breast cancer for 1995/1996 was 202, compared with 411 cases in 2005/2006. This represents a greater than two-fold increase over a decade.

**Table 1:** Demographic details and tumour characteristics of the patients

	**1995/1996**	**2005/2006**	**p-value**
**New cases**	202	411	
**Gender: females**	99%	99%	*NS (t-test)*
**Mean age (range)**	58 years (26-93)	58 years (27-93)	*NS (t-test)*
**Tumour size (mean)**	28.5 mm (SD 22.6)	25.66 mm (SD 22.1)	*NS (t-test)*
**Tumour type**	Invasive 94%	Invasive 87%	*NS (chi square)*
	DCIS 6%	DCIS 13%	
**Invasive subtype**	Ductal 80%	Ductal 75%	
	Lobular 15%	Lobular 14%	*NS (chi square)*
	Other 5%	Other 11%	

(NS = nonsignificant)

The diagnostic modalities utilised in the diagnosis of breast cancer has changed dramatically over the last decade. In 1995/1996, 93% [n = 188] of patients underwent a surgical excision biopsy under general anaesthetic in order to achieve the diagnosis. In contrast, in 2005/2006, 91% [n = 374] of our patients had a preoperative diagnosis established with a tru-cut or core biopsy, which was performed under local anaesthetic in the outpatients department. This refinement towards less invasive diagnostic modalities was statistically significant [χ^2 ^= 0.0001].

**Figure 1:**
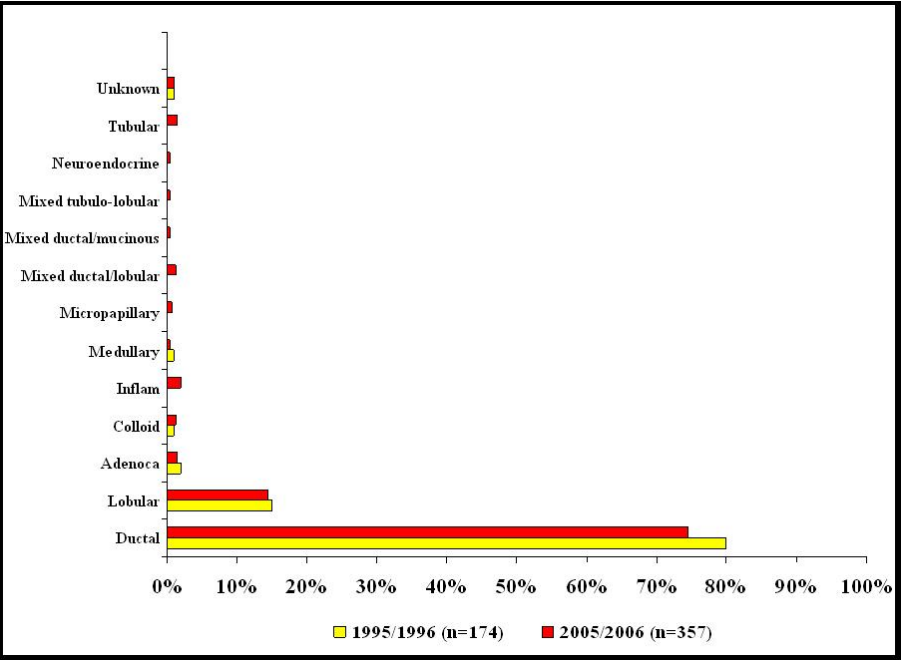
**Invasive histological subtype at diagnosis for new breast cancer patients in 1995/1996 [n = 174] compared with 2005/2006 [n = 358]**.

The tumour type seen at time of diagnosis was similar in both groups - invasive carcinoma was identified in 86% [n = 174] of patients in 1995/1996 and in 87% [n = 358] in 2005/2006. A preoperative diagnosis of ductal carcinoma in-situ [DCIS] was made in 13% of patients in 2005/2006 whereas in-situ carcinoma was only documented in 6% of patients from 1995/1996. The invasive histological subtypes are illustrated in Figure 1.

The most dramatic metamorphosis observed was in respect to patients' primary therapeutic surgical procedure. In 1995/1996, 85% [n = 172] of patients underwent a primary mastectomy compared to only 32% [n = 131] of patients in 2005/2006 [χ^2^*< 0.00005]*. Conversely in 1995/1996, only 15% [n = 30] underwent breast conserving surgery compared to 68% [n = 280] of patients in 2005/2006. Management of the axilla also underwent dramatic revolution, towards a less invasive approach. In 1995/1996 the axilla was managed primarily with a formal axillary clearance for all patients [n = 202] whereas in 2005/2006 61% [n = 250] of patients underwent a sentinel node biopsy [SNB]. More recently, a primary axillary clearance was only performed where there was preoperative radiological or histological confirmation of metastatic disease. If the SNB was positive for macroscopic disease, an axillary clearance was undertaken. In the 2 year period [2005/2006] only 32% [n = 80] of the SNB population had a positive biopsy, necessitating axillary clearance.

Increasing awareness and recognition of DCIS, coupled to patients expectations for good cosmetic outcome following oncological breast surgery, has driven the introduction of immediate breast reconstruction in our unit. The period 2005/2006 saw 148 reconstructive procedures performed, the majority of which were latissimus dorsi flap reconstructions [n = 95]. As breast reconstruction following oncological surgery only came in vogue in the mid 1990's, allied to the paucity of evidence to support its routine use, no reconstructions were performed in our unit in 1995/1996.

**Figure 2:**
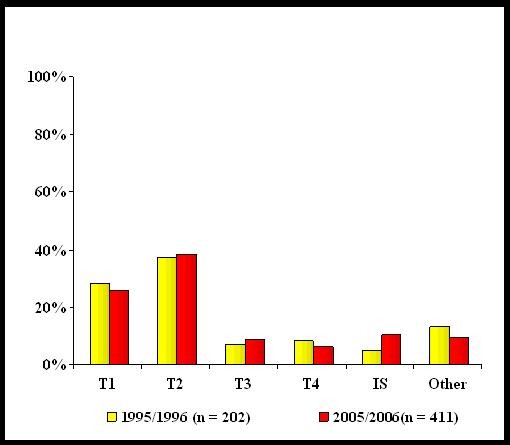
**Tumour size at diagnosis for breast cancer diagnosed in 1995/1996, compared with tumour size in 2005/2006**. [T1: < 2 cm, T2: 2-5 cm, T3: > 5 cm, T4: any size with distant spread, I-S: in-situ carcinoma, Other: size not possible to determine].

Despite efforts to facilitate early diagnosis, our data show no change with respect to stage of disease at presentation over the last decade [Table 1]. Mean tumour size did not change significantly; 28.5 mm in 1995/1996 versus 25.6 mm in 2005/2006, p *= 0.133 *[Figure 2]. The proportions of T1 and T2 tumours in 1995/1996 were 28.5% and 37.5% respectively compared with 26% and 38.5% for 2005/2006 [p = 0.306]. There was a trend towards fewer patients having node positive disease at diagnosis - 56% in 1995/1996 versus 52% in 2005/2006 though this decrease was not reach statistical significance [p = 0.195]. However, a greater proportion of patients were identified as having distant metastatic disease at presentation in the 2005/2006 period; 10.5% [n = 43] compared with only 4.5% [n = 9] in 1995/1996 [p = 0.031]. This likely reflects improvements in diagnostic radiology.

Finally, the last decade has witnessed a transformation in our use of adjuvant treatments for breast cancer. With recent data supporting a disease-free and overall survival benefit associated with adjuvant chemotherapy, particularly for younger patients, a greater proportion of our patient population are having adjuvant therapy. Forty-two [42%] percent of our patients in 2005/2006 received adjuvant chemotherapy versus 32% in 1995/1996 [p < 0.001]. The last decade has seen the introduction of neo-adjuvant chemotherapy in our unit for breast cancer patients and in 2005/2006 7% [n = 29] received preoperative chemotherapy in an attempt to facilitate less radical surgical resection, or downstage the disease. Similarly, the use of local radiotherapy has increased over the last decade, from 17% to 74% [p < 0.0001], reflecting its recommended use in the adjuvant setting in conjunction with minimally invasive surgical resection.

## Discussion

Accurate preoperative diagnosis and staging of breast cancer is of critical importance to facilitate a tailored surgical approach for each individual patient. The European Society for Surgical Oncology guidelines a decade ago recommended that the majority [more than 70%] of breast cancers should receive a pre-operative diagnosis by fine needle cytology or core biopsy [2]. Until the mid 1990's the gold standard diagnostic modality in breast cancer was a surgical excision biopsy. This involved day case admission, a general anaesthetic and a surgical scar. Since the conception of automated core biopsy guns in the mid 1990s, needle core biopsy has been established as a highly accurate diagnostic modality, supplanting the requirement for excision biopsy [4]. It is more cost effective, less invasive and therefore less traumatic to the patient, requires only a local anaesthetic, and can be performed routinely in the outpatient department as part of the triple assessment protocol [5,6]. Core biopsy is currently the gold standard diagnostic technique utilized in our unit and our results show that over the last decade we have adapted best practice recommendations in achieving a preoperative diagnosis with core biopsy for 91% of patients.

For over 80 years, Halsted's radical mastectomy was the surgical treatment of choice for breast cancer, irrespective of tumour size, type or patient age [7]. The late 1970's represented the embryonic stage of breast conserving cancer surgery, with the phenomenon of breast conserving surgery providing adequate oncological resection without compromising patient outcome or survival, being established. Data from randomized controlled trials conducted in the late 1970's and 1980's established equivalent efficacy of breast conserving surgery, in conjunction with radiotherapy, when compared with the traditional radical mastectomy, for the treatment of patients with early stage invasive breast cancer [Stage I and II] [8-11]. Henceforth, the recommended treatment approach to early breast cancer was lumpectomy with a margin of normal breast tissue, followed by local radiotherapy. This was shown to reduce local recurrence rates, though had no effect on long term survival when compared to the traditional surgical approach [12-14]. These evidence based recommendations set a new international standard for breast cancer management [15]. This study illustrates how these recommendations have influenced practice in our unit over the last decade, leading to revolutionary changes in our approach to management of breast cancer patients. We have witnessed a major metamorphosis in diagnostic modalities and primary therapeutic approaches, from traditional radical techniques to a less invasive approach overall. In keeping with this change to breast conserving surgery, the use of local radiotherapy in our unit has increased significantly over the last ten years.

Historically, breast cancer treatment was associated with significant psychosocial morbidity. Clinicians and patients currently place greater emphasis and importance on the cosmetic, psychological, social and emotional outcomes following breast cancer surgery [16]. Many data have shown that immediate breast reconstruction [IBR] is associated with reduced psychological and sexual morbidity, improved physical and emotional outcomes and overall improved quality of life compared to patients who undergo mastectomy without reconstruction [17,18]. Consequently IBR following mastectomy has evolved in our unit, in appropriately selected patients requiring mastectomy as the primary therapeutic procedure, and currently 67% of women requiring a mastectomy undergo IBR.

Adjuvant therapy for breast cancer (hormonal treatments, radiotherapy, and systemic chemotherapy) has also undergone dramatic transformation over the past decade. In 2000, the Dutch National Breast Cancer Platform [NABON] and the Dutch Society for Medical Oncology [NVMO] published a comprehensive consensus statement on adjuvant systemic therapy [19,20]. It recommended tamoxifen use for patients with node-positive tumours and a positive oestrogen receptor [ER] and/or progesterone receptor [PR]. Chemotherapy is recommended for all node-positive tumours in premenopausal women, and in postmenopausal women under age 70 who had negative hormonal receptor status. For node-negative tumours, the recommendation of adjuvant therapy also depends on the tumour size, differentiation grade and mitotic activity index. Prior to the introduction of the Dutch guidelines, tamoxifen was recommended for postmenopausal women with node-positive, ER positive tumours while chemotherapy was reserved for premenopausal women with node-positive tumours. Our data reflects the uptake of these recommendations for appropriate use of systemic therapies over the last decade.

## Conclusion

This study has demonstrated that radical changes have occurred in the management of breast cancer in the last decade. The current minimally invasive diagnostic procedures and availability of sophisticated imaging techniques facilitate the planning of accurate individual management regimens preoperatively. Breast conserving surgery with adjuvant radiotherapy and immediate breast reconstruction has been shown not only to be safe and effective but also to reduce patients' associated psychosocial morbidity. Similarly minimal axillary surgery has been shown to provide the necessary prognostic information regarding management of the axilla, obviating the need for routine axillary clearance with its associated morbidity. Breast cancer management has undergone a seismic revolution in the last decade with immense benefit to the patient, and it is encouraging to see how we have remained abreast of new developments in breast cancer management over that time. It remains incumbent upon us to continue to adapt our practice patterns in light of emerging knowledge. There is need for continuous reviews of breast cancer literature, consequent revisions of management guidelines, and audit of practice patterns, to ensure that quality care continues to be delivered in accordance with best available evidence.

## Abbreviations

WLE: Wide local excision; SNB: Sentinel Node Biopsy; DCIS: Ductal carcinoma in-situ; IBR: Immediate breast reconstruction; ER: Estrogen receptor. PR: Progesterone receptor.

## Competing interests

The authors declare they have no competing interests.

## Authors' contributions

HMH was primarily responsible for literature search, collation of data, statistical analyses, drafting, and submission and the manuscript. RSP participated in the design of the study, drafting and revising the manuscript. AD was involved in data collection and statistical analysis. CM and RM contributed to the patient database and clinical management of patients. KJS and MJK conceived of the study, participated in its design, directed clinical management of the patients, and reviewed the manuscript. All authors read and approved the final manuscript.

## Authors' information

HMH is a surgical research fellow, RSP a Specialist Registrar in General/Breast/Endocrine surgery, AD is a medical student, CM and RM are consultant general and breast surgeons in Galway University Hospital. KJS is a consultant *BreastCheck *and general surgeon, at Galway University Hospital. MJK - a Breast/Endocrine Surgeon - is the Professor of Surgery at Galway University Hospital and a Principal Investigator with the Health Research Board in Ireland.

## Pre-publication history

The pre-publication history for this paper can be accessed here:


